# Flash Glucose Monitoring and Diabetes Mellitus Induced by Immune Checkpoint Inhibitors: An Approach to Clinical Practice

**DOI:** 10.1155/2022/4508633

**Published:** 2022-11-04

**Authors:** Pablo Rodríguez de Vera-Gómez, Ana Piñar-Gutiérrez, Raquel Guerrero-Vázquez, Virginia Bellido, Cristóbal Morales-Portillo, María Pilar Sancho-Márquez, Pablo Espejo-García, Noelia Gros-Herguido, Gema López-Gallardo, María Asunción Martínez-Brocca, Alfonso Soto-Moreno

**Affiliations:** ^1^Endocrinology and Nutrition Department, Hospital Universitario Virgen Macarena, Seville, Spain; ^2^Endocrinology and Nutrition Department, Hospital Universitario Virgen del Rocío, Seville, Spain; ^3^Oncology Department, Hospital Universitario Virgen del Rocío, Seville, Spain; ^4^Oncology Department, Hospital Universitario Virgen Macarena, Sevilla, Spain

## Abstract

**Objectives:**

The aim of this study is to investigate in depth diabetes mellitus associated with immune checkpoint inhibitors (DM-ICIs) by analysing a case series. We also evaluated the clinical impact of flash glucose monitoring (FGM) systems in the management of this entity.

**Methods:**

We conducted an observational cohort study of DM-ICIs diagnosed in two hospitals in Seville (Spain). Patients with a new diagnosis of diabetes mellitus (DM) or with sudden worsening of preexisting DM after starting treatment with ICIs, with a random 5 hour-postprandial C-peptide value of <0.6 nmol/L and without possibility of subsequent withdrawal of insulin treatment, were included.

**Results:**

A total of 7 cases were identified, mostly males (*n* = 6; 85.7%), with a mean age of 64.9 years. The mean glycated hemoglobin (HbA1c) upon diagnosis was 8.1%, with diabetic ketoacidosis (DKA) observed in 6 cases (85.7%). Subcutaneous flash glucose monitoring (FGM) systems were used in six cases, with a mean follow-up period of 42.7 weeks. During the first 90 days of use, mean average glucose was 167.5 mg/dL, with a coefficient of variation (CV) of 34.6%. The mean time in the range 70-180 mg/dL (TIR) was 59.7%, with a mean time above range (TAR) 181-250 mg/dL of 27.8% and a mean TAR > 250 mg/dL of 10.2%. The mean time below range (TBR) 54-69 mg/dL was 2%, while the mean TBR < 54 mg/dL was 0.3%. The mean glucose management indicator (GMI) was 7.3%. No significant differences were observed in FGM values for the following 90 days of follow-up. A progressive improvement in all parameters of glycaemic control was observed between the first month of FGM use and the sixth month of FGM use. Of note, there was a decrease in mean CV (40.6% to 34.1%, *p* = 0.25), mean TAR 181-250 (30.3% to 26%, *p* = 0.49), mean TAR > 250 mg/dL (16.3% to 7.7%, *p* = 0.09), mean TBR 54-69 mg/dL (5.2% to 2%, *p* = 0.16), and mean TBR < 54 mg/dL (1.8% to 0.2%, *p* = 0.31), along with an increase in mean values of TIR 70-180 mg/dL (46.5% to 60.5%, *p* = 0.09). The lack of statistical significance in the differences observed in the mean FGM values over the follow-up period may be related to the small sample size.

**Conclusion:**

DM-ICI is recognised by a state of sudden-onset insulinopenia, often associated with DKA. The use of FGM systems may be a valid option for the effective management of DM-ICIs and for the prevention of severe hyperglycaemic and hypoglycaemic episodes in this condition.

## 1. Introduction

In the last few years, immune checkpoint inhibitors (ICIs) have positioned themselves as one of the therapeutic options with the greatest benefits in the treatment of different types of cancer. Their favourable effects have been proven in a wide variety of clinical scenarios, including an increase in survival in advanced metastatic tumours such as advanced melanoma [1, 2]. Consequently, ICI use in clinical practice is becoming increasingly widespread in healthcare systems around the world.

From a biomolecular point of view, ICIs act by blocking inhibitory pathways in T lymphocytes (T cells) mediated by CTLA-4 (cytotoxic T-lymphocyte antigen 4), PD-1 (programmed cell death protein-1), and PD-L1 (programmed death ligand 1), with the aim of avoiding immune tolerance and increasing the T-cell ability to identify and respond to tumour cells [3]. As a consequence of this mechanism, immune tolerance processes in healthy tissues may be altered, leading to autoimmune phenomena that are termed immune-related adverse events (irAEs). These autoimmune phenomena can lead to endocrinopathies such as hypothyroidism and hyperthyroidism, hypophysitis, adrenal insufficiency, generalised lipodystrophy, or insulinopenic diabetes [4, 5].

Diabetes mellitus associated with immune checkpoint inhibitors (DM-ICIs) is a rare condition in clinical practice. Its incidence is estimated to be around 1-2% among patients receiving treatment with ICIs, although an upward trend has been observed in recent years in relation to the increased presence of these drugs in cancer treatment regimens [6, 7]. From a pathophysiological perspective, DM-ICI is due to a massive and fulminant immune-mediated destruction of pancreatic *β*-cells, as a consequence of overactivation of T cells (mostly CD8+) in the context of treatment with ICIs [8]. This mechanism involves a sudden development of a state of insulinopenia, usually accompanied by hyperglycaemic crises often associated with diabetic ketoacidosis (DKA) and with islet autoantibody positivity ranging from 20% to 71% [8, 9]. Interestingly, the fulminant variant of type 1 diabetes mellitus (T1DM) described in Asian populations is characterised by a pattern similar to that of DM-ICIs, with a sudden onset and the common presence of markers of exocrine pancreatic damage, so both conditions may share similar pathophysiological mechanisms [10, 11].

Currently, knowledge about DM-ICIs is scarce, given the low number of available cases due to the exceptional nature of its onset. The identification of risk factors for its development or its association with a favourable tumour response has been the subject of recent research [12, 13]. However, aspects such as the description of the clinical course of this diabetes type in the medium/long-term remains scarcely studied.

In addition, the potential severity associated with this condition and the expected increase in its incidence as a consequence of the increasing indications for ICIs in cancer treatment regimens raise the need for the development of specific strategies for prevention and management of DM-ICIs [14].

In this sense, the implementation of new technological tools such as flash glucose monitoring (FGM) tools could have a positive impact on patients with DM-ICIs, especially in the prevention of hypoglycaemia and severe hyperglycaemic crisis. In the International Time in Range Consensus, Battelino et al. have proposed glycaemic control targets based on metrics from interstitial glucose monitoring systems, such as a glycaemic variability target (as defined by a coefficient of variation (CV) ≤ 36%) or different percentages of time in glycaemic ranges, for 14-day periods of use with collection of more than 70% of the data [15]. However, to our knowledge, to date, the benefits of FGM systems and the validity of these glycaemic targets in patients with DM-ICIs have not yet been explored.

In this paper, we report a series of seven cases of DM-ICIs diagnosed in two hospitals located in Spain, with special emphasis on the benefits of the use of FGM systems for the management of this condition.

## 2. Methods

We conducted a retrospective observational study of DM-ICI cases referred during the 2018-2021 period to the Endocrinology and Nutrition Departments of University Hospital Virgen Macarena and University Hospital Virgen del Rocío in Seville (Spain).

The following criteria were used to diagnose cases of DM-ICIs: (1) diagnosis of diabetes after receiving the first course of treatment with ICIs, (2) onset with an episode of acute hyperglycaemia ≥ 200 mg/dL or glycated hemoglobin (HbA1c) ≥ 6.5% (taking into account that in case of sudden presentation, HbA1c may not be elevated at the time of diagnosis), (3) demonstration of *β*-cell destruction as evidenced by a result < 0.6 nmol/L in a random 5 h postprandial C-peptide measurement (as it has been suggested by the ADA-EASD consensus report for the management of T1DM) [8, 16].

Patients with a history of type 2 diabetes (T2DM) or prediabetes who had an episode of acute deterioration of glucose control after starting treatment with ICIs, with evidence of newly developed insulinopenia associated with the need for insulin therapy, were also included [17].

The state of glucose control prior to the development of DM-ICIs was assessed by baseline glycaemia levels recorded in routine monthly blood tests requested by the Oncology Center, together with HbA1c levels (determined by high-performance liquid chromatographic method) in case these where available. After diagnosis of DM-ICIs, islet autoantibodies (anti-GAD65 and anti-IA-2 autoantibodies) were measured by electrochemiluminescence immunoassay (ECLIA, Roche). Where available, human leukocyte antigen- (HLA-) II-DQ genotyping was determined by polymerase chain reaction and sequence specific oligonucleotide probe (PCR-SSOP), using the INNO-LIPA HLA-DQ model (Fujirebio, Spain).

Initially, follow-up of glycaemic control after diagnosis of DM-ICIs was performed by self-monitoring of blood glucose (SMBG) through glucometers in all cases. Subsequently, subcutaneous flash glucose monitoring (FGM) systems (FreeStyle Libre 2®, Abbott Laboratories) were implemented in those cases in which it was technically possible, analysing glucose data from 30-day and 90-day ambulatory glucose profile (AGP) reports.

Quantitative data were expressed as mean, standard deviation (SD), median, and range (minimum-maximum). For the assessment of the evolution of AGP data, a repeated measures general linear model with Bonferroni correction was developed. The significance level was defined by a *p* value < 0.05, considering *p* values < 0.1 as close to statistical significance. The statistical analysis software SPSS v.26 (IBM Statistics) was used to perform the statistical analysis.

This study was conducted in accordance with the ethical standards proposed in the Declaration of Helsinki (1964) and in its subsequent modifications. Data were collected retrospectively as part of routine patient care, in accordance with the guidelines of the hospitals' Ethics Committees.

## 3. Results

A total of seven cases were identified. The overall characteristics of the study cohort are summarised in Table 1, while the individual patient characteristics are summarised in Table 2.

85.7% of the cases were male (*n* = 6), and the overall mean age was 64.9 years (SD: 15.2, median: 71 years, range: 38-82 years). The mean body mass index was 26.11 kg/m^2^ (SD: 3.9, median: 25.9 kg/m^2^, range: 22.1-30.2 kg/m^2^). The cancer diagnosis was melanoma in 42.9% of cases (*n* = 3, one of them being uveal melanoma), lung adenocarcinoma in 28.55% (*n* = 2), and urothelial carcinoma in 28.55% (*n* = 2). One case (14.3%) received treatment with Ipilimumab (anti-CTLA-4)/nivolumab (anti-PD1), while five cases (71.4%) received monotherapy with nivolumab, pembrolizumab, cetrelimab (anti-PD1 drugs), and one case (14.3%) with atezolizumab (anti-PD-L1). A personal history of T2DM was recorded in one case (14.3%) and prediabetes in another case (14.3%), while the remaining patients (*n* = 5, 71.4%) had no history of diabetes.

The mean number of weeks from ICIs treatment initiation to diagnosis of DM-ICs was 15.8 weeks (SD: 14.7, median: 8.14 weeks, range: 3.6-45 weeks). The mean number of ICI treatment cycles administered prior to diagnosis was 4.3 (SD: 2.6, median: 3 cycles, range: 2-8 cycles).

Out of the 7 cases, six had DKA at diagnosis, while one had isolated hyperglycaemia (Table 1). Mean glycaemia at diagnosis was 668 mg/dL (SD: 278.5, median: 572 mg/dL, range: 350-1078 mg/dL). Markedly elevated values of pancreatic lipase were observed in 2 out of 5 cases (patient 2 and patient 7), in whom this parameter was available, with a decrease to the normal range after resolution of the hyperglycaemic episode that led to the diagnosis of DM-ICIs. In these two cases, there was no clinical evidence of malabsorption, nor were there any changes in imaging tests (abdominal CT) suggesting acute pancreatitis.

**Figure 1:**
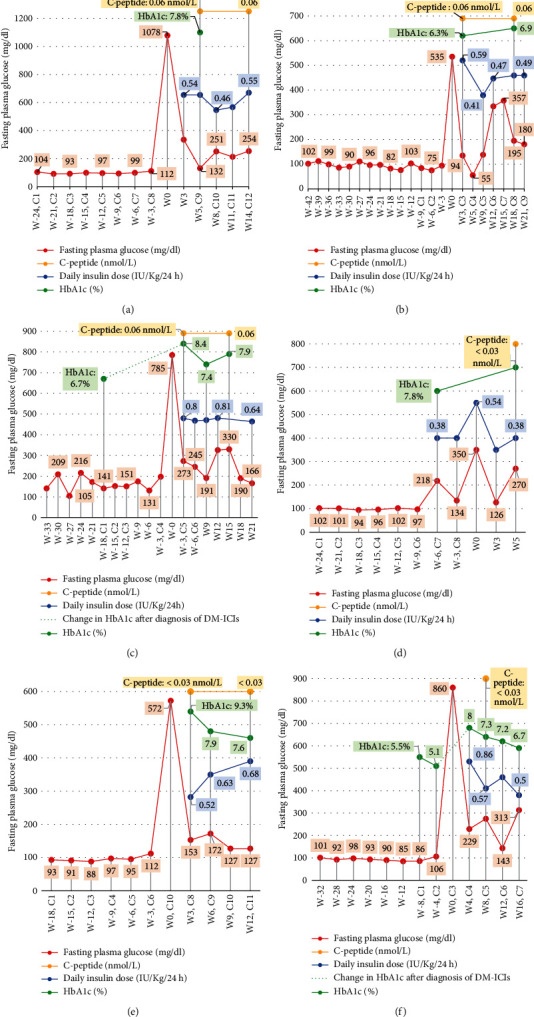
Markers of glucose homeostasis and beta-cell function in patients with DM-ICIs. Abbreviation list: HbA1c: glycated hemoglobin. On horizontal axis: Cx: number of treatment cycles with ICIs; Wx: number of weeks of follow-up from DM-ICIs diagnosis. C-peptide: random 5 h post-meal measurement. (a) Patient 1, (b) patient 2, (c) patient 3, (d) patient 4, (e) patient 5, (f) patient 6.

**Figure 2:**
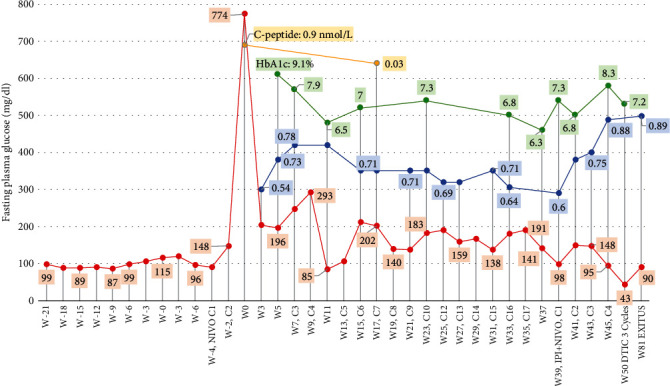
Markers of glucose homeostasis and beta-cell function in patient 7. Abbreviation list: DTIC: dacarbazine; HbA1c: glycated hemoglobin; IPI + NIVO C1: first cycle of the ipilimumab + nivolumab scheme; NIVO C1: first course of treatment with nivolumab. On horizontal axis: Cx: number of treatment cycles; Wx: number of weeks of follow-up from DM-ICIs diagnosis. C-peptide: random 5 h postmeal measurement.

Blood tests performed prior to the diagnosis of DM-ICIs as part of the usual cancer follow-up revealed an abnormal increase in fasting plasma glucose (between 106 and 148 mg/dL) in 4 out of 7 cases with respect to what these patients had shown in previous follow-up visits (Figures 1(a), 1(e), and 1(f) and Figure 2). In the other cases, no alteration in plasma glucose levels preceding the DM-ICIs diagnosis was observed.

Mean HbA1c value at debut was 8.1% (65 mmol/mol), measured at a mean of 20 days after diagnosis of DM-ICIs. The C-peptide level was below 0.6 nmol/L in all cases, with a mean of 43.5 days comprised between DM-ICIs diagnosis and C-peptide measurement. C-peptide measurement was a random measurement performed within 5 hours postmeal, coinciding with blood glucose values between 100 and 200 mg/dL in all cases. In this regard, case 7 initially showed a detectable C-peptide level (0.9 nmol/L; 12 days after DM-ICIs diagnosis) which subsequently decreased to insulinopenic levels (0.03 nmol/L at 28 weeks after DM-ICIs diagnosis) (Figure 2).

All cases required subcutaneous basal-bolus insulin regimen for the management of DM-ICIs. The overall mean insulin requirement amounted to 0.6 IU/Kg body weight/24 hours (SD: 0.13), remaining constant throughout the follow-up period, with no decrease observed during the first weeks after the onset of DM-ICIs (Table 1). In two cases, high-dose corticosteroid treatment was prescribed after DM-ICI diagnosis (methylprednisolone 80 mg/24 h), but no remission of the condition was observed.

Moreover, 5 out of the 7 cases (71.4%) showed single islet autoantibody positivity (*n* = 2 anti-GAD65+, *n* = 3 anti-IA2+), while the other two cases (28.6%) were islet autoantibody-negative. The HLA-II DQ genotype was explored in *n* = 3 cases, with two of them showing the DQA1^∗^05 : 01/DQB1^∗^02 : 01 haplotype (DQ2.5 haplotype; patient 1 and patient 3; Tables 1 and 2), which confers a high risk for the development of T1DM [18].

The mean follow-up period during which the patients were monitored after the DM-ICIs diagnosis was 71.3 weeks (SD: 44.5, median: 67 weeks, range: 27-145 weeks) (Table 1).

In 6 out of 7 cases (patients 1-6; 85.7%), FGM systems were used as part of the glycaemic control optimisation strategy, activating audible and vibrating alarms for hypoglycaemia (<70 mg/dL) and hyperglycaemia (>250 mg/dL). Initiation of use of these devices occurred after a mean of 28.7 weeks from diagnosis of DM-ICIs (SD: 35.7 weeks) and continued for a mean of 42.7 weeks thereafter (SD: 20.3 weeks) (Tables 1 and 3). The remaining patient (patient 7) used daily capillary blood glucose monitoring as a method of glycaemic monitoring.

AGP reports were obtained from each patient's personal device. To assess the evolution of glycaemic control during follow-up, 30-day glycaemic monitoring data were obtained in the first, third, and sixth month from the start of FGM use. In addition, to assess the overall glycaemia of each participant, glycaemic data were extracted for 180 days of FGM use, in two consecutive 90-day periods (Table 3).

**Figure 3:**
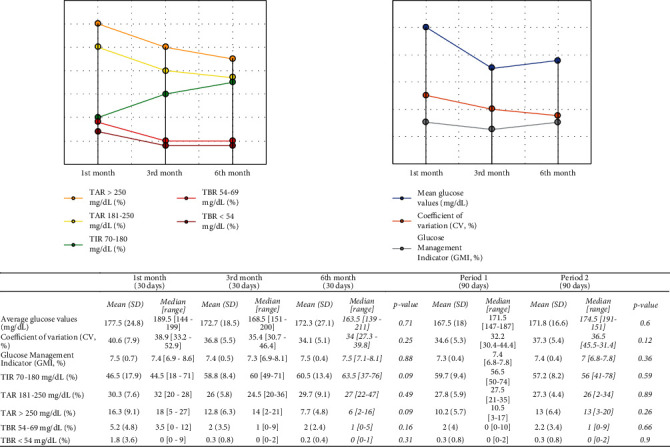
Mean flash glucose monitoring data from the DM-ICI cohort. Abbreviation list: TIR: time in range; TAR: time above range; TBR: time below range; GMI: glucose management indicator; CV: coefficient of variation. Data are shown as mean ± standard deviation. For all cases, values are presented as mean values related to FGM use of 30 days (1st, 3rd, and 6th months) and to FGM use of 90 days (periods 1 and 2).

Figure 3 shows the overall blood glucose data of the DM-ICIs cohort. During the first 90 days of follow-up, the mean glycaemia was 167.5 mg/dL (SD: 18 mg/dL, median: 171.5 mg/dL, range: 147-187 mg/dL), with a mean CV of 34.6% (SD: 5.3%; median: 32.2%, range: 30.4-44.4 mg/dL) and a mean glucose management indicator (GMI) of 7.3% (SD: 0.4%, median: 7.4%, range: 6.8-7.8%). The mean time in range (TIR) 70-180 mg/dL was 59.7% (SD: 9.4%, median: 56.5%, range: 50-74%), while the mean time above range (TAR) 181-250 mg/dL was 27.8% (SD: 5.9%, median: 27.5%, range: 21-35%), and the mean TAR > 250 mg/dL was 10.2% (SD: 5.7%; median: 10.5%, range: 3-17%). Mean time below range (TBR) 54-69 mg/dL was 2% (SD: 4%, median: 0%, range: 0-10%), and mean TBR < 54 mg/dL was 0.3% (SD: 0.8%, median: 0%, range: 0-2%). No significant differences were obtained with respect to the subsequent 90-day period (Figure 3).

**Figure 4:**
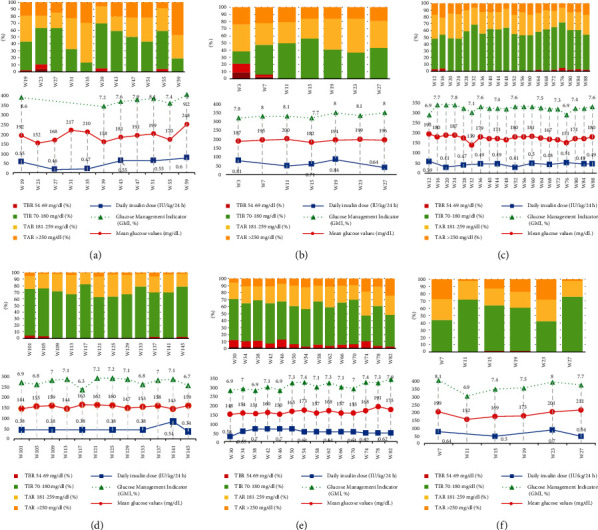
Summary of ambulatory glucose profile (AGP) reports obtained from flash glucose monitoring (FGM) devices in patients with DM-ICIs. Abbreviation list: Cx: number of treatment cycles with ICIs; GMI: glucose management indicator; TAR: time above range; TBR: time below range; TIR: time in range; Wx: number of weeks of follow-up from DM-ICIs diagnosis. Report (a) patient 1, (b) patient 2, (c) patient 3, (d) patient 4, (e) patient 5, and (f) patient 6. Patient 7 did not use FGM devices. In each graph, the upper part of the image shows the percentage of time spent in different glucose ranges, whereas the lower part of the image shows mean glucose values (mg/dL), glucose management indicator (GMI, %), and daily insulin dose (IU/Kg/24 h). Each vertical line on the horizontal axis shows 30 days of MFG use data from each patient's AGP report.

Analysing the FGM data through the monthly AGP reports, we observed a progressive improvement in all parameters of glycaemic control from the first month of use of the FGM devices (Figure 4). Thus, CV (which is a marker of glucose variability) decreased from an initial mean of 40.6% (SD: 7.9%) to 34.1% (SD: 5.1%) on the sixth month of use (although without reaching statistical significance, *p* value = 0.25), along with TAR > 250 mg/dL (mean 16.3% on the first month vs. 7.7% on the sixth month, *p* value = 0.09).

Consistently, there was an increase in TIR between the first and the sixth month of FGM use (46.5% vs. 60.5%, *p* value = 0.09) as well as a decrease in TBR 54-69 mg/dL (5.2% vs. 2%, *p* value = 0.16) and TBR < 54 mg/dL (1.8% vs. 0.2%, *p* value = 0.31) (Figure 3).

The percentage of time FGM sensor was active was higher than 80% in all cases at all timepoints, thus supporting a correct interpretation of FGM data in our cohort. Indeed, according to the International Consensus on Time in Range [15], the percentage of time the glucose sensor has to be active in order to properly interpret the continuous glucose monitoring (CGM) data is >70% (from 14 days of sensor use).

Five patients (71.4%) discontinued ICI treatment after diagnosis of DM-ICIs, while 2 (28.6%) patients continued the scheduled treatment regimen. Of the five patients who discontinued treatment, one did so permanently. Four patients resumed ICI treatment after a mean of 14 weeks (SD: 6.8 weeks, median: 12.8 weeks, range: 7.4-23.1 weeks), and no deterioration in glucose control was observed in any of the cases (who remained on insulin therapy).

Once the diagnosis was made, coordinated clinical visits were scheduled between Oncology and Endocrinology Centers for close clinical follow-up. The possibility of an unscheduled clinical assessment at the Diabetes Day Hospital of the Endocrinology Department was made available in the case there were alterations in glycaemic control requiring preferential or urgent clinical assessment.

Four participants developed other irAEs in addition to DM-ICIs, the most common being vitiligo (*n* = 2; Table 2). In addition, two cases had other immune-related endocrinopathies (one case of hypophysitis with secondary adrenal insufficiency and one case of primary autoimmune hypothyroidism). Reevaluation of cancer response to ICI treatment showed complete response in *n* = 3 cases (42.8%), stable disease in *n* = 2 cases (28.6%), partial response in *n* = 1 case (14.3%), and disease progression (with exitus) in *n* = 1 case (14.3%, Table 2).

## 4. Discussion

In this paper, we describe a cohort of seven cases of DM-ICIs of whom we retrieved glucose control data during follow-up from FGM systems. To the best of our knowledge, this is the first study that incorporates this tool in patients with DM-ICIs, which allows a preliminary assessment of the impact of the use of new technologies in this population.

In our series, the mean age at diagnosis was 64.9 years, consistent with previously published data and in contrast to T1DM, whose onset occurs mostly during infancy and childhood [19, 20]. The time from ICI treatment initiation to diagnosis of DM-ICIs was heterogeneous, with a mean of 15.8 weeks, a minimum of 3.6 weeks, and a maximum of 45 weeks. These data are consistent with those reported in major published cohorts, where a mean of 9 weeks to diagnosis was observed, with atypical cases ranging from a few days after the first administration of ICIs until years after completion of treatment [8, 21].

In relation to the treatment used, in our study, most cases received PD-1/PD-L1 inhibitor regimens in monotherapy or in combination, the latter regimen being the most frequently associated with the development of DM-ICIs [8, 22]. In this regard, it has been reported that in nonobese diabetic (NOD) mice (an animal model of T1DM), PD-1/PD-L1 blockade is associated with rapid disease development [23]. In addition, it has been described how *β*-cells from healthy individuals express PD-L1 in their membrane as a protective mechanism against immune attacks, suggesting the relevant role of PD-L1 pathway in pancreatic *β*-cell survival [24].

DM-ICIs is characterised by a state of insulinopenia with a sudden onset, leading to a high percentage of cases developing DKA at the time of clinical presentation (more than 67%) [8, 21]. In our cohort, 85.7% of cases (*n* = 6) had DKA at diagnosis.

Based on this mechanism, a C-peptide measurement below 0.6 nmol/L has been proposed as a diagnostic criterion for DM-ICIs by most authors [8, 12–14]. However, this cut-off point differs from that proposed by the American Diabetes Association (ADA) and the European Association for the Study of Diabetes (EASD) in their consensus document for the diagnosis of T1DM, where a random C-peptide value (measured within 5 hours after meal) of <0.6 nmol/L (without concomitant hypoglycaemia or long fasting period) is considered suggestive of insulinopenic DM [16]. This may lead to additional difficulty in the diagnosis of those cases of insulinopenic DM-ICIs with random C-peptide values within the lower cut-off point (e.g., between 0.4 and 0.6 nmol/L). In our view, a clear definition of the diagnostic value of C-peptide indicative of DM-ICIs (e.g., <0.2 nmol/L, as it has been observed in our cohort) could homogenize the diagnosis of this rare condition. In this sense, in our case series, all patients presented a C − peptide value ≤ 0.06 nmol/L (in patient 7, this value was detected upon a second late measurement, specifically 98 days postdiagnosis), confirming the absence of endocrine pancreatic reserve (Table 2).

Data from the FGM devices show that suboptimal control with a tendency to hyperglycemic episodes was maintained throughout the follow-up period, without a decrease in daily insulin requirements, which supports the theory of massive and persistent pancreatic *β*-cell destruction in DM-ICIs. In addition, some studies have suggested that DM-ICIs may be characterised by dysregulations of pancreatic *α*-cells and incretin system, with implications for *α*-cell glucagon production and/or incretin secretion (e.g., reduced glucagon-like peptide 1 secretion) [8, 25]. This could lead to a greater tendency to glycaemic variability with an increased risk of hypoglycaemia, although the evidence available so far is limited [8, 26].

In DM-ICIs, HbA1c levels are often not markedly high as compared to the observed glucose values that sometimes are extremely high at the time of diagnosis [27]. This finding reflects the sudden onset of DM-ICIs. In our study, mean HbA1c value was moderately high (8.1%), probably as a consequence of its delayed measurement (2-3 weeks, on average, after DM-ICIs diagnosis). On the other hand, HbA1c may have limitations when reporting the degree of glucose control of patients with DM-ICIs, as it has interferences with other diabetes-independent factors such as anemia, chronic kidney disease, or periodic red blood cell transfusions (frequent in cancer patients) [28]. In this regard, none of the patients in our cohort were identified as having any of these factors interfering with the HbA1c measurement.

The development of DM-ICIs in patients with a previous history of T2DM constitutes an entity with particular characteristics, with some cases reported in the current literature [8]. However, no homogeneous criteria for diagnosis of DM-ICIs in the context of T2DM have been formulated. Some of the proposed criteria are the worsening of glycaemic control (in terms of HbA1c) with the need for insulin therapy, evidence of seroconversion with islet autoantibodies positivity or new development of insulinopenia with undetectable or reduced C-peptide values [8]. Nevertheless, in patients with insulinopenic T2DM or poor metabolic control, the diagnosis of DM-ICIs could be challenging, with the additional difficulty of other factors such as the islet autoantibody positivity rate being less than 50% in this condition [10, 17, 29]. In our study, we recorded one case with preexistent T2DM and another with a previous diagnosis of prediabetes. Although we did not have a baseline C-peptide measurement, once the event (DM-ICIs) has developed, we detected C-peptide levels within the insulinopenic range. The diagnosis was made following a sudden episode of acute hyperglycaemic crisis requiring insulin treatment [21].

In our cohort, in one case (patient 7), C-peptide measurement during the first 24 hours after DM-ICIs diagnosis showed a normal value (0.9 nmol/L), which fell into the insulinopenic range in a subsequent measurement. In this regard, some authors propose a second confirmatory C-peptide measurement at least 15 days after DM-ICI diagnosis, or measurement under glucagon stimulation or with a mixed meal tolerance test in case of doubt [30]. The decrease in C-peptide at such an early stage is a remarkable difference between DM-ICIs and T1DM, where periods of several years with residual C-peptide are frequently observed, and presence of islet autoimmunity (as evidence by islet autoantibody positivity) is present in approximately 80-90% of patients [8, 31, 32].

In our research, 71.4% of cases (*n* = 5) were positive for islet autoantibodies, a percentage that is slightly higher than that reported in the main reports on DM-ICIs, which place it at around 50% [8]. This is another relevant difference compared to T1DM, where islet autoantibody positivity is higher than 90% and is considered a diagnostic criterion [20]. The observed data suggest a secondary role of humoral immunity in the pathogenesis of DM-ICIs, where impaired T-lymphocyte regulation is predominant and islet autoantibodies may not mediate cytotoxic action [21, 33]. However, the role of these antibodies in DM-ICIs remains unclear, partly due to the absence of studies reporting the serological status prior to the development of this condition. Some authors have formulated the hypothesis that baseline islet autoantibody positivity could act as a risk factor for DM-ICIs [8]. However, islet autoantibody measurement is not currently considered a cost-effective strategy for DM-ICI screening, largely because of its low prevalence in the general population (around 1.7%) [19, 34].

In the present case series, two cases (out of three studied) had the haplotype DQA1^∗^05 : 01/DQB1^∗^02 : 01 (DQ2.5 haplotype) within the genetic risk assessment for the development of autoimmune diabetes [18]. This haplotype is frequently associated with DRB1^∗^03 binding imbalances (thus creating the haplotype called DR3-DQ2) and, like other HLA class II polymorphisms such as DR4-DQ8 (DRB1^∗^04-DQA1^∗^03-DQB1^∗^03 : 02), has been associated with a strong risk for T1DM development [17, 18, 35]. In this regard, a similar prevalence of DR4 has been reported in patients with DM-ICIs and T1DM (around 50% of cases), suggesting that both conditions possibly share genetic risk factors [21, 36]. However, other authors have also described an overrepresentation of T1DM protective haplotypes in patients with DM-ICIs (such as DR7, DR11 o DR15), which casts doubt on the real role of genetic predisposition in the pathogenesis of DM-ICIs [21]. This position is reinforced by the fact that DR9 (an HLA-II haplotype associated with Asian Fulminant Diabetes (AFD), which has a similar course to that of DM-ICIs) has not been found to be overrepresented [19, 20, 37].

Some authors have suggested the existence of different subtypes of DM-ICIs depending on the clinical and biochemical profile at the onset [25]. Thus, the majority of patients exhibit a clinical course similar to that of AFD, with a sudden onset characterised by DKA and dysfunction in the secretion of counter-regulatory hormones (such as glucagon due to involvement of pancreatic *α* cells), together with impaired exocrine pancreatic function [21, 26]. In contrast, another subset of patients follows a course more closely resembling that of typical T1DM, with a longer time between the clinical onset and the development of a state of irreversible insulinopenia, and with the absence of a global pancreatic involvement. The existence of a third group with mixed features has also been suggested [21, 25]. The basis for these differences may lie in the individual profile of predisposition to the development of autoimmune diabetes. Thus, it has been reported that patients without islet autoantibodies, or with T1DM-protective HLA-II haplotypes, tend to develop DM-ICIs later, suggesting a modulatory role of these variables [8].

In line with this insight, in our series, 4 cases (57.1%) showed features compatible with a course similar to that of AFD, while 2 cases showed mixed features (negative islet autoantibodies) and 1 case showed a slower development time, with an onset in the form of isolated hyperglycaemia without DKA. Interestingly, the AGP reports extracted from the FGM devices showed how the patient diagnosed following an episode of isolated hyperglycaemia (patient 4) had a better glucose control compared to the other patients during postonset follow-up, with the highest TIR 70-180 mg/dL and the lowest CV (as compared to the other cases) and with a TBR < 70 mg/dL of less than 1.5%. Assuming the limitations derived from the analysis of a single case, this fact would support the theory of the existence of different onset forms of DM-ICIs, including one that shares clinical features comparable to those of typical T1DM.

Different strategies have been proposed for the early identification of DM-ICIs. Some of them are providing health education notions focused on the identification of warning symptoms or the design of care circuits for specific assessment. In those cases, in the presence of a history of established T2DM or prediabetes, it is recommended to optimise glucose control [8–11, 19]. In patients with risk factors for the development of autoimmune diseases (personal or family history), implementing the pre-ICI-treatment study with islet autoantibody measurement or HLA class II genotyping, or providing the patient with a glucometer for SMBG, may be valid options to consider in order to identify high-risk patients who are candidate for closer clinical monitoring [8, 19].

Periodic blood tests performed at the same time of the treatment cycle administration may be useful to identify discrete variations in glycaemia levels that precede the debut of DM-ICIs [19]. In our study, in 4 cases (57.1%), hyperglycaemia was recorded in the blood test corresponding to the treatment cycle prior to the diagnosis of the condition, so that the detection of such alterations should prompt an early diabetologist evaluation to prevent the development of acute metabolic decompensation.

On the other hand, Fujiwara et al. have described changes in insulin secretion dynamics after initiation of treatment with ICIs in patients who subsequently developed DM-ICIs [38], so that C-peptide monitoring in at-risk patients may be a strategy to consider. HbA1c monitoring during the pre-DM-ICIs period is not recommended, given the tendency to normal values for this parameter at the time of diagnosis (related to the abrupt onset of the condition) [8]. Cases have been reported with the development of DM-ICIs two years after discontinuation of treatment with ICIs, so it is recommended that glycaemia levels continue to be monitored even after discontinuation of treatment [19, 27].

In the event of a hyperglycaemic crisis in a patient being treated with ICIs, a differential diagnosis with similar conditions should be considered [39]. The presence of concomitant treatment with glucocorticoids or other hyperglycaemia-inducing drugs (such as phosphoinositide 3-kinase inhibitors) should be explored [19]. In these cases, preserved C-peptide levels, absence of islet autoantibodies, and/or the possibility of withdrawing insulin therapy after blood glucose stabilisation can assist the differential diagnosis [8, 19]. Although elevated values of markers of exocrine pancreatic injury have been described in cases of DM-ICIs, the presence of acute abdominal computed tomography (CT) changes, or the occurrence of steatorrhoea or malabsorption can be indicative of acute pancreatitis as a trigger for hyperglycaemic crisis [40]. On the other hand, within endocrine irAEs, cases of generalised immune-mediated lipodystrophy have been described, which could justify the occurrence of severe hyperglycaemia in patients treated with ICIs. In these cases, generalised alterations of subcutaneous fat tissue or the presence of hypertriglyceridaemia and hyperglycaemia secondary to a sharp increase in insulin resistance (with elevated C-peptide values) are the most common features [21, 41].

In terms of initial therapeutic management, the use of corticosteroids or other immunomodulators has not been shown to be effective in the treatment of DM-ICIs due to the frequent irreversibility of insulin secretion dysfunction, unlike other nonendocrine irAEs [3, 42]. A correct diagnosis to allow early initiation of treatment and initiation of a period of diabetes education has been proposed as the main measures in the initial management of DM-ICIs [19, 21].

On the other hand, given the difficulty of managing this condition, close and coordinated follow-up with other specialties could have added advantages in optimising the metabolic control of patients, allowing complications to be resolved at an early stage and progressively increasing the degree of autonomy in the management of diabetes. In this sense, multidisciplinary collaboration between different specialties (including oncologists and endocrinologists) plays a key role [42].

The complexity of clinical management and adjustment of insulin therapy in DM-ICIs makes early implantation of continuous glucose monitoring systems a very useful tool in patients with this condition. In our case series, the benefits associated with FGM resulted in a progressive and generalised improvement in glucose parameters between the first and subsequent months of FGM use. In this regard, the CGM metrics observed decrease in CV and TBR < 70 mg/dL and TBR < 54 mg/dL was noteworthy, which is remarkable given the risk factors for the development of hypoglycaemia associated with this condition (sudden-onset insulinopenia with subsequent need for titration of insulin therapy, impaired release of the counterregulatory hormones, age, and additional comorbidities). We also observed a progressive increase in TIR 70-180 mg/dL, which overall was higher than 54% during the entire follow-up; also, we observed a decrease in TAR > 250 mg/dL from 16.3% in the first month to 7.7% in the sixth month. These differences failed to reach statistical significance (*p* value > 0.05), probably due to the low number of cases in our cohort as a consequence of the rarity of this diabetes type. Yet, the increase in TIR and the decrease in TAR > 250 were close to statistical significance (*p* value in 0.05-0.1 range), suggesting that with a larger sample size, these differences could become statistically significant.

Furthermore, there were no hospital readmissions for acute hyperglycaemic crises. Sensor usage data showed proper patient use of FGM systems. In this sense, factors, such as the presence of warning alarms, or trend arrows are FGM resources that could be associated with the achievement of these favourable results.

Currently, no specific glycaemic targets have been formulated for patients with DM-ICIs. Prioritising the prevention of severe hypoglycaemias and hyperglycaemias with associated life-threatening conditions or the need for hospital admission is considered to be one of the fundamental therapeutic goals [43]. The FGM data reported in this case series demonstrate how the recommendations issued in the “International Consensus on Time in Range” for high-risk patients could be reasonable targets for patients with DM-ICIs. For high-risk patients (those with a higher risk of complications, those with comorbid conditions, or those requiring assisted care), guidelines issued by the International Consensus on Time in Range propose as acceptable targets a TBR < 70 mg/dL less than 1%, a TBR < 54 mg/dL close to 0%, a TIR 70-180 mg/dL greater than 50%, and a TAR > 250 mg/dL < 10% [15]. However, endpoints in each patient should be approached on an individualised basis, taking into account aspects such as the cancer-specific survival perspective or the burden of comorbidities.

The potential use of other diabetes technologies such as continuous subcutaneous insulin infusion systems or hybrid-closed loop systems is options that have not been explored in patients with DM-ICIs but could have added benefits in certain patient subgroups.

We acknowledge that the present study has some limitations. First, the small number of patients we described (due to the rarity of DM-ICIs) may limit the interpretation of our study conclusions, requiring comparison of our results with other similar cohorts. Second, the lack of measurement of C-peptide and islet autoantibodies at baseline limits the interpretation of the process through which DM-ICIs develop. The measurement of zinc transporter 8 autoantibodies (ZnT8A; considered one of the main islet autoantibodies) was not available in our setting, nor was the measurement of the HLA-DR haplotypes. HLA-DQ haplotypes could only be determined in three patients due to factors external to the investigation. On the other hand, use of FGM systems was relatively delayed after the disease onset; therefore, FGM use may not entirely reflect the exact patterns of blood glucose derangements in our cohort. In addition, longer-term follow-up of patients with DM-ICIs (and ideally after discontinuation of treatment with ICIs) would confirm the theory of a persistent insulin secretion dysfunction that cannot be reversed.

In conclusion, DM-ICI is a condition characterised by a high complexity in terms of management, in which an early diagnosis allows the prompt initiation of insulin treatment and the use of technological systems (such as FGM devices) that represent strategies leading to significant clinical benefits, including improvement of glucose control, prevention of hypoglycemic episodes, and reduction of hospital readmissions. In this regard, the presence of an endocrinologist in the multidisciplinary team around the cancer patients is a crucial aspect since it facilitates the implementation of coordinated strategies during the follow-up.

Therefore, large prospective studies using continuous glucose monitoring systems are needed to better investigate the patterns of glucose derangements in patients with DM-ICIs and to understand the most effective therapeutic interventions in this population.

## Figures and Tables

**Table 1:** Main characteristics of the DM-ICI study cohort.

Total number of cases	*n* = 7
Gender	Male 85.7% (*n* = 6)/female 14.3% (*n* = 1)
Age	64.9 ± 15.2 (minimum: 38; maximum: 82)
BMI (kg/m^2^)	26.1 ± 3.9 (minimum: 22.1; maximum: 30.2)
Weeks between ICI treatment initiation and DM-ICI diagnosis	15.8 ± 14.7 (minimum: 3.6; maximum: 45)
Number of ICI treatment cycles before DM-ICIs	4.3 ± 2.6 (minimum: 2; maximum: 8)
Debut with DKA	85.7% (*n* = 6)
Increased markers of exocrine pancreatic injury	40% (*n* = 2, study available in 5 cases)
Peak blood glucose detected (mg/dL)	668 ± 278.5 (minimum: 350; maximum: 1078)
HbA1c (%) at diagnosis	8.1 ± 1.1 (minimum: 6.3; maximum: 9.3)
C-peptide (nmol/L)	0.05 ± 0.1 (minimum: 0.01; maximum: 0.28)
Islet autoantibody positivity	71.4% (*n* = 5)
GAD65 antibody positivity	28.6% (*n* = 2)
IA2 antibody positivity	42.9% (*n* = 3)
DQA1^∗^05 : 01/DQB1^∗^02 : 01 haplotype	*n* = 2, HLA genotyping available in 3 cases
Insulin requirements (average over total follow-up, IU/kg/day)	0.6 ± 0.13 (minimum: 0.42; maximum: 0.73)
Other irAEs	57.1% (*n* = 4)
Interruption of ICI treatment (weeks without treatment)	71.4% (*n* = 5); 14 ± 6.8 (minimum: 7.4; maximum: 23.1)
Total follow-up time since diagnosis DM-ICIs (weeks)	71.3 ± 44.5 (minimum: 27; maximum: 145)
Users of FGM systems	85.7% (*n* = 6)
Weeks between DM-ICI diagnosis and first use of FGM	28.7 ± 35.7 (minimum: 3; maximum: 101.1)
Total weeks of FGM use	42.7 ± 20.3 (minimum: 20; maximum: 76)

Abbreviation list: BMI: body mass index; DM: diabetes mellitus; DM-ICIs: diabetes mellitus related to immune checkpoint inhibitors; DKA: diabetic ketoacidosis; FGM: flash glucose monitoring; HLA: human leukocyte antigen; GAD65: glutamic acid decarboxylase 65-kilodalton isoform; IA2: islet antigen 2; irAEs: immune-related adverse events. Data has been registered at the time of diagnosis of DM-ICIs. Quantitative data are expressed as mean ± standard deviation (SD) (minimum-maximum).

**Table 2:** Anthropometric, clinical, and laboratory characteristics of patients with DM-ICIs.

	Patient 1	Patient 2	Patient 3	Patient 4	Patient 5	Patient 6	Patient 7
Gender	M	F	M	M	M	M	M
Age (years)^∗^	82	38	77	72	59	71	55
Race	Caucasian	Caucasian	Caucasian	Caucasian	Caucasian	Caucasian	Caucasian
BMI (kg/m^2^)^∗^	29.9	22.1	22.5	22.2	30	25.9	30.2
History of autoimmune diseases	No	Primary autoimmune hypothyroidism	No	No	No	No	No
History of DM	No	No	T2DM (treated with metformin)	No	No	No	Prediabetes (treated with nutrition therapy)
Cancer diagnosis (TNM)	LADC (T4N2M1a)	Uveal melanoma (T1N0M1a)	LADC (T1N0M1a)	Melanoma (T2N1M0)	Urothelial carcinoma (T2N2M0)	Urothelial ca (T2NxM0)	Melanoma (T4N0M0)
ICI	Atezolizumab (anti-PD-L1)	Ipilimumab/nivolumab (anti-CTLA-4/PD-1)	Pembrolizumab (anti-PD-1)	Nivolumab (anti-PD-1)	Pembrolizumab (anti-PD-1)	Cetrelimab (anti-PD-1)	Nivolumab (anti-PD-1)
Weeks to DM-ICIs (no. cycles)^1^	23.1 (8)	7.1 (2)	8.1 (3)	45 (6)	18 (7)	5.7 (2)	3.6 (2)
Clinical manifestation at the onset of DM-ICIs	DKA	DKA	DKA	IH	DKA	DKA	DKA
Acute kidney injury at the onset of DM-ICIs	Yes	No	Yes	No	No	Yes	Yes
Pancreatic lipase (U/L). Reference range: 12-70	N.A.	723	13	N.A.	42	72	805
Peak blood glucose detected (mg/dL)	1078	535	785	350	572	860	774
HbA1c, % (days between the diagnosis of DM-ICIs and HbA1c measurement)	7.8 (42)	6.3 (14)	8.4 (14)	7.8 (7)	9.3 (21)	8 (28)	9.1 (14)
C-peptide^2^, nmol/L (days between the diagnosis of DM-ICIs and C-peptide measurement); reference range: 0.5-1.5	0.06 (42)	0.06 (14)	0.06 (14)	0.03 (84)	0.03 (21)	0.03 (32)	0.03 (98)
Islet autoantibodies							
Anti-GAD65 (U/mL) ref.: 0-17	5.8	5.9	146.6	6.7	8.7	7	>280
Anti-IA2 (U/mL) ref.: 0-27	6.9	96.9	7.2	37.4	28.9	12.9	2.7
HLA class II (DQ) haplotypes	DQA1^∗^05 : 01/DQB1^∗^02 : 01^†^; DQA1^∗^02 : 01/DQB1^∗^03 : 01	DQA1^∗^01 : 02/DQB1^∗^06 : 04; DQA1^∗^01 : 04/DQB1^∗^05 : 01	DQA1^∗^05 : 01/DQB1^∗^02 : 01^†^; DQA1^∗^01 : 01/DQB1^∗^05 : 01	N.A.	N.A.	N.A.	N.A.
Daily insulin requirements (mean over the entire follow-up period, expressed as IU/kg/24 hours)	0.54	0.73	0.48	0.42	0.73	0.7	0.56
Discontinuation of ICI treatment after DM-ICI diagnosis (weeks without treatment)	No	Yes (14.9)	Yes (7.4)	No	Yes (10.7)	Yes (no restart)	Yes (23.1)
Continuation of ICI treatment after the achievement of glycemic targets (total ICI treatment cycles received)	Yes (21)	Yes (9)	Yes (6)	Yes (13)	Yes (33)	No (2)	Yes (16)
FGM system user	Yes	Yes	Yes	Yes	Yes	Yes	No
Other irAEs (grade^3^)	No	Diarrhoea (G2), vitiligo (G1), immune-mediated cystitis (G3)	Diarrhoea (G1), pancytopenia (G3)	Hypophysitis with secondary adrenal insufficiency (G3), subacute cutaneous lupus erythematosus (G2), vitiligo (G1)	Primary autoimmune hypothyroidism (G1)	No	No
Tumour response to ICIs	Stable disease	Partial response	Stable disease	Complete response	Complete response	Complete response	Disease progression (exitus)
Total follow-up time (weeks from the diagnosis of DM-ICIs)	59	27	88	145	82	27	67

Abbreviation list: BMI: body mass index; CTLA-4: cytotoxic T-lymphocyte antigen 4; DKA: diabetic ketoacidosis; DM: diabetes mellitus; DM-ICIs: diabetes mellitus related to immune checkpoint inhibitors; F: female; FGM: flash glucose monitoring; GAD65: glutamic acid decarboxylase 65-kilodalton isoform; HbA1c: glycated hemoglobin; HLA: human leukocyte antigen; IA2: islet antigen 2; ICI: immune checkpoint inhibitor; IH: isolated hyperglycaemia; irAEs: immune-related adverse events; LADC: lung adenocarcinoma; M: male; N.A.: not available, PD-1: programmed cell death protein-1; PD-L1: programmed death ligand 1; T2DM: type 2 diabetes mellitus. ^∗^At the time of diagnosis of DM-ICIs. ^†^DQA1^∗^05 : 01/DQB1^∗^02 : 01 haplotype (DQ2.5 haplotype, which is recognised as a haplotype associated with a high for development of type 1 diabetes). ^1^Weeks and number of ICIs treatment cycles between the start of ICI treatment and the diagnosis of DM-ICIs. ^2^C-peptide: random and postprandial measurement within 5 h postmeal. ^3^Degree of severity calculated using the Common Terminology Criteria for Adverse Events (CTCAE), in line with ESMO guidelines [42].

**Table 3:** Flash glucose monitoring (FGM) data of the study participants during the follow-up period^1,2,3^.

	Follow-up	Data period	Average glucose	Coefficient of variation (%)	GMI (%)	TIR 70-180 mg/dL	TAR 181-250 mg/dL	TAR >250 mg/dL	TBR 54-69 mg/dL	TBR <54 mg/dL
Patient 1	Weeks before FGM sensor implant: 19 Follow-up with FGM (weeks): 40	1st month	192	33.9	8.6	42	37	19	2	0
		3rd month	168	35.3	7.2	62	22	15	1	0
		6th month	158	39.8	7.6	65	24	6	5	0
		Period 1-3 months	162	30.4	7.2	68	27	5	0	0
		Period 3-6 months	191	37.6	7.9	48	31	20	1	0
Patient 2	Weeks before FGM sensor implant: 3 Follow-up with FGM (weeks): 24	1st month	187	52.9	7.8	18	38	24	12	9
		3rd month	200	35.4	8.1	50	29	21	0	0
		6th month	199	27.3	8.1	37	47	16	0	0
		Period 1-3 months	181	31.3	7.6	53	34	13	0	0
		Period 3-6 months	170	35.3	7.4	61	27	11	1	0
Patient 3	Weeks before FGM sensor implant: 12 Follow-up with FGM (weeks): 76	1st month	195	45.7	6.9	45	35	17	3	0
		3rd month	189	30.7	7.8	49	36	15	0	0
		6th month	139	33.3	7.1	68	25	6	1	0
		Period 1-3 months	187	31.8	7.8	50	35	15	0	0
		Period 3-6 months	186	32.5	7.8	51	34	15	0	0
Patient 4	Weeks before FGM sensor implant: 101.1 Follow-up with FGM (weeks): 44	1st month	144	33.2	6.9	71	20	5	4	0
		3rd month	159	33.2	7	71	26	2	1	0
		6th month	162	29.7	7.2	62	31	6	1	0
		Period 1-3 months	147	32.6	6.8	74	21	3	2	0
		Period 3-6 months	153	31.4	7	71	25	3	1	0
Patient 5	Weeks before FGM sensor implant: 29.9 Follow-up with FGM (weeks): 52	1st month	148	42.5	6.9	59	23	6	10	2
		3rd month	151	46.4	6.9	58	20	11	9	2
		6th month	165	39.6	7.3	55	29	10	5	1
		Period 1-3 months	147	44.4	6.8	58	22	8	10	2
		Period 3-6 months	152	45.5	6.9	57	22	10	9	2
Patient 6	Weeks before FGM sensor implant: 7.3	1st month	199	35.2	8.1	44	29	27	0	0
		3rd month	169	39.5	7.4	63	23	13	1	0
	Follow-up with FGM (weeks): 24	6th month	211	34.6	7.7	76	22	2	0	0
		Period 1-3 months	181	37.1	7.6	55	28	17	0	0
		Period 3-6 months	179	41.2	7.6	55	25	19	1	0

Abbreviation list: FGM: flash glucose monitoring; GMI: glucose management indicator; TAR: time above range; TBR: time below range; TIR: time in range. ^1^Data were obtained from the ambulatory glucose profile (AGP) reports for each patient. ^2^AGP reports have been set to show data for 30 days of FGM use for the first, third, and sixth month of FGM use, and for 90 days of FGM use for the two consecutive periods “Period 1-3 months” and “3-6 months.” ^3^The percentage of time FGM sensor was active was higher than 80% in all cases at all timepoints.

## Data Availability

Individual clinical, laboratory, and flash glucose monitoring data used to support the conclusions of this study have not been made available due to the need to preserve the publication of personal data of the patients included in this research.
